# Comparison of Short and Standard Implants in the Posterior Mandible: A 3D Analysis Using Finite Element Method

**Published:** 2018-03

**Authors:** Allahyar Geramy, Amirreza Rokn, Abbasali Keshtkar, Abbas Monzavi, Hamid Mahmood Hashemi, Tahereh Bitaraf

**Affiliations:** 1 Professor, Dental Research Center, Dentistry Research Institute, Tehran University of Medical Sciences, Tehran, Iran; Department of Orthodontics, School of Dentistry, Tehran University of Medical Sciences, Tehran, Iran; 2 Professor, Dental Implant Research Center, Dentistry Research Institute, Tehran University of Medical Sciences, Tehran, Iran; Department of Periodontics, School of Dentistry, Tehran University of Medical Sciences, Tehran, Iran; 3 Assistant Professor, Department of Health Sciences Education Development, School of Public Health, Tehran University of Medical Sciences, Tehran, Iran; 4 Professor, Dental Implant Research Center, Dentistry Research Institute, Tehran University of Medical Sciences, Tehran, Iran; 5 Professor, Dental Implant Research Center, Dentistry Research Institute, Tehran University of Medical Sciences, Tehran, Iran; Department of Oral and Maxillofacial Surgery, School of Dentistry, Tehran University of Medical Sciences, Tehran, Iran; 6 PhD Student, Dental Implant Research Center, Dentistry Research Institute, Tehran University of Medical Sciences, Tehran, Iran

**Keywords:** Dental Implants, Dental Prosthesis Design, Dental Stress Analysis, Finite Element Analysis, Alveolar Ridge Augmentation

## Abstract

**Objectives:**

This study aimed to analyze functional stresses around short and long implant-supported prostheses with different crown heights.

**Materials and Methods:**

Four three-dimensional (3D) models were designed with SolidWorks 2015. In models 1 (control) and 2, three dental implants (second premolar 4.1×8 mm, molars: 4.8×8 mm) were placed. In models 3 and 4, three dental implants (second premolar 4.1×4 mm, molars: 4.8×4) were placed. Residual bone height was 10 mm in groups 1 and 2 (grafted bone) models and 6 mm in groups 3 and 4. The crown heights were modeled at 11.5 mm for groups 1 to 3, and 15 mm for group 4. The applied oblique force was 220 N to simulate chewing movements. The maximum von Mises and principal stresses on the implants and the supporting tissues were compared using the 3D finite element method.

**Results:**

In all models, the highest stress value was seen within the most coronal part of bone (crestal bone), which was cortical or grafted bone. The highest stress values in the bone supporting the implant neck were seen in the premolar region of each model, especially in model 4 (291.16 MPa). The lowest stress values were demonstrated in the molar region of model 3 (48.066 MPa). The model 2 implants showed the highest von Mises stress concentrated at their neck (424.44 MPa).

**Conclusions:**

In atrophic posterior mandible with increased crown height space, short implants with wider diameter seem to be a more feasible approach compared to grafting methods.

## INTRODUCTION

Dental implants are considered as a common treatment option to replace the missing teeth with reliable and safe long-term outcomes. However, insufficient bone volume is a critical factor against achieving the optimal treatment result. Bone grafts and short dental implants have been proven to be efficient for rehabilitation of atrophic edentulous areas such as atrophic posterior mandible due to the presence of the inferior alveolar nerve in this region [1–3]. Short dental implants have been proposed as a simpler, cheaper and faster alternative for rehabilitation of atrophic edentulous areas to avoid disadvantages of the surgical techniques such as high technical sensitivity and postoperative complications [1,2]. The definition of short dental implants is still controversial in previous researches regarding the cut-off length between short and standard implants. Dental implants with intra-bony lengths of less than 10, 8 or 7 mm are defined as short implants in different studies. Current clinical literature considers 7 mm long or shorter implants as “short” or “extra-short” dental implants [1,2]. Four-millimeter dental implant, which is the shortest marketed dental implant, has been evaluated by some studies in terms of its biomechanics [1,4–7].

Comparison between short and standard implants in native or augmented bone may affect their outcomes. In some studies, short implants have been associated with decreased success rates when compared to longer implants in sufficient bone. However, the outcome of short implants compared to standard implants in the absence of adequate bone is more reasonable because dentists tend to place longer implants in sufficient bone [8,9].

Increased crown height space caused by bone loss may increase prosthetic complications but not influencing the clinical performance of implants. Some biomechanical studies stated that high crown height prosthesis could be an unfavorable factor for stress distribution. The crown height space more than 15 mm determined in biomechanical studies is dangerous [10,11]. In addition, crown-to-implant ratio of 2:1 is accepted by the Academy of Osseointegration [11].

The purpose of this study was to illustrate, using three-dimensional finite element method, a possible difference in stress distribution in 4 mm implants and longer implants supporting fixed partial dentures with variable crown heights, and their surrounding tissues (cortical, cancellous and grafted bones). The null hypothesis was that short implants would have similar risk of bone loss to conventional implants in augmented bone, and increasing the crown height would not adversely affect stress distribution in the peri-implant bone.

## MATERIALS AND METHODS

**Fig. 1:**
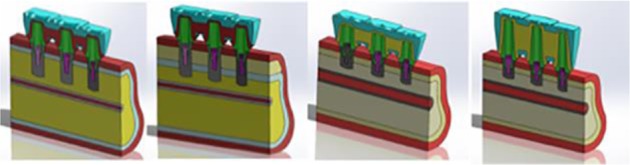
Cross-sectional view of three-dimensional computer- aided design models of the supporting tissues (blue: cortical bone, yellow: cancellous and grafted bone), implants (grey), and prosthetic structures [abutment screw (violet), metal frame (red), porcelain (blue)]. Left to right: Three-dimensional structures of model 1, model 2, model 3 and model 4, respectively. (a): 8 mm cancellous bone, (b): 2 mm cortical bone, (c): 4 mm cancellous bone, (d):4 mm grafted bone, and (e): all models had at least 6.8 mm bone width

The present study simulated a condition in which the mandibular second premolar and first and second molars were missing and replaced with fixed partial denture supported by three dental implants. For the current research, four three-dimensional (3D) models were designed (Fig. 1). A model of the posterior atrophic mandible was constructed via cone beam computed tomography images that were obtained for implant treatment planning of an actual patient. The mandibular bone was modeled showing cortical and trabecular bone, presenting properties of type II bone [12] in SolidWorks 2015 software (SolidWorks Corp., Waltham, MA, USA). Residual bone height was 10 mm in model 1 and it was 6 mm in the other three models. In model 2, grafted bone (4 mm in height) was applied on the bone simulating guided bone regeneration procedure [13].

**Fig. 2:**
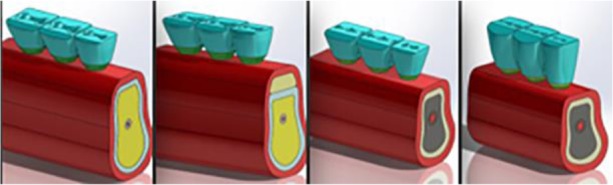
Three-dimensional merging of prosthetic structures and supporting tissues. The connectors were 6 mm in height and 5 mm in width for model 1 to 3 (left to right), and 9×5 mm for model 4

All models had at least 6.8 mm bone width and 2 mm cortical bone thickness. All dental implants and abutment designs were simplified using SolidWorks 2015, but they maintained their similarity to real models of Straumann Company (SLActive Roxolid Standard Plus Implants with Regular CrossF it connection, 033.561S, 033.591S, 033.043S, 033.044S, and RN synOcta Meso abutment 048.560). In models 1 (as control) and 2, three dental implants (second premolar 4.1×8 mm, molars: 4.8×8 mm) and their abutments were placed into the prepared models. In models 3 and 4, three dental implants (second premolar 4.1×4 mm, molars: 4.8×4) and their abutments were placed in the reconstructed models. The height of splinted metal-ceramic bridge was modeled at 11.5 mm for model 1 to 3, and 15 mm for the last model (Figs. 1 and 2). For the crown models, a model of average-sized mandibular second premolar and molars was prepared [6].

The models were meshed between 151,624 and 628,801 nodes and 77,991 to 432,055 elements. All mechanical properties of each simulated material were adopted from previous researches and are summarized in Table 1 [5,6,10,14]. With regard to boundary and loading conditions, the buccal and lingual base of each model were restrained, while 220 N oblique occlusal force was applied to the central fossa of the premolar and molars in lingualbuccal direction. ANSYS Workbench 11 software (ANSYS Inc., Canonsburg, USA) was utilized for stress analysis, and maximum von Mises stress and principal stress in each model were reported. Each model was divided into the following subsections to evaluate the stresses. The maximum von Mises stress and principal stress (in the buccal and lingual sides) of the cortical, cancellous and grafted bone and the dental implants were determined.

**Table 1.** Mechanical properties of the materials modeled

	**Young’s modulus (GPa)**	**Poisson’s ratio**
Implant and abutment (Ti-6Al-4V^*^)	110	0.35
(Ti-Zr^**^ alloy)	100	0.3
Cortical bone (2 mm)	13.7	0.26
Trabecular bone	1.37	0.38
Graft material (completely matured)	3.45	0.31
Co-Cr^***^ structure	218	0.33
Feldspathic porcelain	82.8	0.35
Mucosa (2 mm)	19.6	0.30
Resin	7	0.2

* Ti-6Al-4V: Titanium-aluminum-vanadium

** Ti-Zr: titanium-zirconium

*** Co-Cr: Cobalt-chromium

In this research, the stress values of the areas that increased ultimate compressive or tensile stress in the bone were compared between the models.

## RESULTS

Maximum von Mises and principal stress values calculated in each model are illustrated in Table 2. Compared to the maximum principal and von Mises stresses in the supporting tissues of all models, the highest stress value was seen within the most coronal part of the bone, the crestal bone, which was either cortical or grafted bone. The stresses in the cortical bone (stiffer bone) were higher than the trabecular bone in all models. According to lingual-vestibular direction of applied force, the maximum principal stress (compressive stress) focused on the buccal side was lower than the maximum principal stress (tensile stress) converged on the lingual bone for all the supporting tissues.

**Table 2.** Maximum values for principal stresses and von Mises stresses for each model

**Treatment model**	**Treatment model**	**Treatment model**	**Maximum principal stress (MPa)**						**Maximum von Mises stress (MPa)**		

			**Lingual**			**Buccal**					

			M2^*^	M1^*^	P2^*^	M2^*^	M1^*^	P2^*^	M2^*^	M1^*^	P2^*^
**Model 1**	4.1 × 8 mm CH^**^: 11.5	Crestal cortical bone	65.35	66.89	58.31	18.69	21.26	19.95	107.66	110.54	96.74
		Trabecular bone	10.98	11.19	11.78	0.16	2.51	0.49	13.76	16.35	14.18
		Standard implant							235.24	216.53	321.65

**Model 2**	4.1 × 8 mm CH: 11.5	Grafted bone	115.01	87.72	96.95	24.29	23.71	27.59	106.85	92.47	117.51
		Crestal cortical bone	3.00	1.91	2.93	5.95	5.68	4.90	12.868	12.008	11.649
		Trabecular bone	1.90	2.00	1.59	0.88	1.68	1.30	3.08	2.40	3.22
		Standard implant							364.02	365.20	424.44

**Model 3**	4.1× 4 mm CH: 11.5	Crestal cortical bone	56.27	59.83	113.28	19.67	17.06	20.35	48.06	43.53	148.56
		Trabecular bone	2.50	4.71	3.81	2.23	3.18	2.77	4.49	6.77	5.32
		Short implant							220.82	210.93	191.67

**Model 4**	4.1× 4 mm CH: 15	Crestal cortical bone	71.13	85.12	191.97	24.33	18.06	27.52	70.17	81.726	291.16
		Trabecular bone	7.81	3.13	8.42	4.31	4.41	4.80	5.92	6.14	8.88
		Short implant							238.35	276.12	209.27

* M2: Second molar, M1: First molar, P2: Second premolar

** CH: Crown height space

**Fig. 3:**
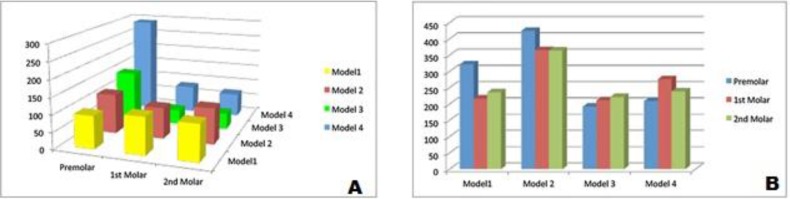
Graphical representations of the maximum von Mises stress according to different treatment designs: (A) in cortical bone ring around the implant neck, (B) in dental implants

**Fig. 4:**
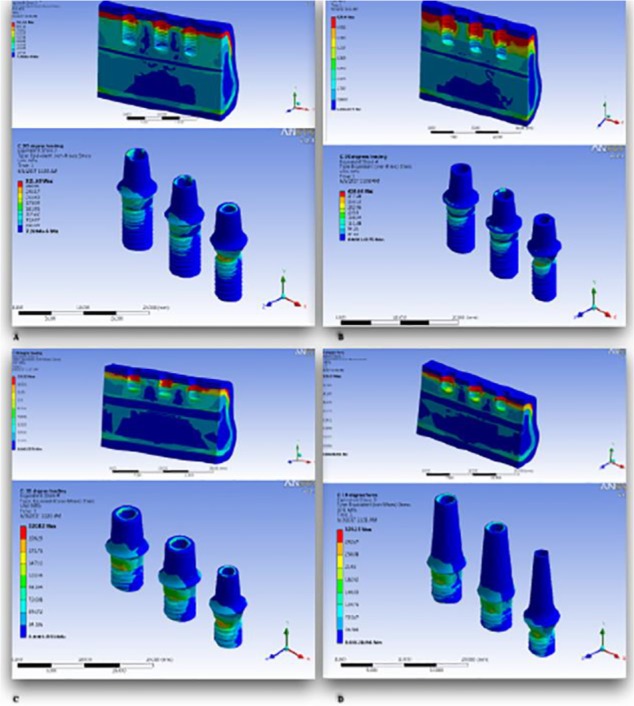
Maximum von Mises stress distribution in the supporting tissues and dental implants of the 4 models [model 1 (A), model 2 (B), model 3 (C), model 4 (D)]

The highest stress values in the cortical bone supporting implant neck were seen in the premolar region of each model, especially in model 4 (291.16 MPa) in which the stress was significantly higher than the molar areas (Fig. 3A). It seems that the maximum von Mises stresses in the cortical bone of premolar regions of model 1 to 3 were not considerably different. The lowest stress values were demonstrated in molar region of model 3 (48.066 MPa), which slightly increased in models 4, 2 and 1, respectively. In model 2, the grafted bone showed higher stress value compared to the cortical bone. The maximum von Mises stresses for dental implants are shown in Table 2 and Fig. 3B. The von Mises stresses were concentrated in the neck of implants in all models (Fig. 4). Compared to the von Mises stress for all implants, the 8 mm implant in model 2 showed the highest stress value (365 and 424.44 MPa).

## DISCUSSION

This study evaluated stress distribution in the posterior mandible for four treatment options (standard implant, standard implant in augmented bone, short implant with normal crown height, and short implant with increased crown height). In the present study, the highest stress value was seen within the coronal part of bone, crestal bone (cortical or grafted bone) in each model. The highest stress values in the bone supporting implant necks were seen in the premolar region of each model, especially in model 4. The lowest stress value was demonstrated in molar region of model 3. The standard implants in model 2 showed the highest von Mises stress concentrated in their neck. According to these findings, it could be interpreted that short implants with wider diameters had similar or better stress concentration in marginal bone compared to the conventional implants, and increasing the crown height may adversely affect stress distribution in the peri-implant bone; therefore, the null hypothesis was not accepted.

Four-millimeter long implant (Q: 4.1 mm) with 15 mm crown height (model 4) revealed the highest stress values in bone; however, 4mm implant (Q: 4.8 mm) with 15 and 11.5 mm crown heights (models 3 and 4) had the lowest stress values in bone. The results of this study indicated that higher stresses occurred in the supporting bone around the 4 mm implant with smaller diameter. Effect of increasing the crown height from 10 to 15 mm was assessed in some finite element studies, and revealed that this factor could enhance stress concentration and displacement in bone mainly under oblique loading. In addition, stress distribution was damaging in implant prosthetic screws [15,16].

The implant survival rates for increased implant-to-crown ratios (0.8:1 to 3.0:1) were recently evaluated and showed outcomes similar to normal bone resorption [17–19]. However, some studies described that increased crown height may cause screw loosening and abutment fracture in the posterior regions of the jaws [20]. The results of our study were in agreement with the afore-mentioned studies since the increased crown height caused higher stress levels within the tolerance of the peri-implants tissues. However, the stress value in the premolar region of cortical bone that exceeded ultimate stress in group 4 was considerably large. According to previous studies, ultimate compressive and tensile stresses were reported to be 170 MPa and 130 MPa respectively, for cortical bone, and 20 MPa for cancellous bone [6, 21,22]. Short implants, which are considered as a minimally invasive approach, could be an alternative treatment for atrophic posterior mandible. However, their long-term prosthetic results remain to be determined [1]. According to the present results, the stress value of crestal bone around short implants was comparable to that of standard implants, especially when the crown height space was not increased.

These data agree with earlier studies that reported short implants to be a feasible approach for the posterior regions of atrophic jaws [1,4–6,8]. In the current study, the maximum von Misses stress in the grafted bone (model 2) was almost similar to the model 1 cortical bone. When the force passed through the grafted bone, the amount of stress in native bone decreased (model 2). The stress levels in the model 2 implants increased considerably within the range of tolerance compared to the other models. Mature grafted bone could lead to a more equitable stress distribution in the multilayer bone surrounding the dental implant [23,24]. However, the stress values in the graft tissue were not desirable in the present study. These data agree with previous studies that revealed that the most coronal 2 to 3 mm part of the implant is responsible for the transmission of maximum load to the supporting tissue, and cortical bone, due to higher modulus of elasticity, bears a greater load than does the trabecular bone [23–25].

Vertical ridge augmentation procedures have been advocated with the understanding of less predictable outcome in an atrophic mandible because of mandibular bone density and composition [13]. Lee et al. [2] stated that survival rates of short implants and longer implants in augmented bone were not significantly different, and short implants could be an alternative treatment to reduce surgical complications.

The 3D finite element method, which is an effective tool for biomechanical analysis, has some limitations related to high sensitivity to the assumptions made regarding model parameters, such as material properties and loading and boundary conditions [10]. Dynamic loading simulations and real bone to implant contact needs to be considered in future investigations.

## CONCLUSION

According to the current finite element study, the short implant with normal crown height space could be the most feasible approach for an atrophic mandible. In an atrophic posterior mandible with increased crown height space, short implants with wider diameters seem to be a simpler approach compared to grafting methods. However, biomechanical benefits of long implants should be considered in cases with bone resorption. Long implants in grafted bone and short implants with increased crown height space could be used for rehabilitation of atrophic mandible.
